# Potential impacts of high-sensitivity creatine kinase-MB on long-term clinical outcomes in patients with stable coronary heart disease

**DOI:** 10.1038/s41598-020-61894-3

**Published:** 2020-03-27

**Authors:** Yen-Wen Wu, Sing Kong Ho, Wei-Kung Tseng, Hung-I Yeh, Hsin-Bang Leu, Wei-Hsian Yin, Tsung-Hsien Lin, Kuan-Cheng Chang, Ji-Hung Wang, Chau-Chung Wu, Jaw-Wen Chen

**Affiliations:** 1 Cardiology Division of Cardiovascular Medical Center, Far Eastern Memorial Hospital, New Taipei City, Taiwan; 2 National Yang-Ming University School of Medicine, Taipei, Taiwan; 3 Department of Medical Imaging and Radiological Sciences, I-Shou University, Kaohsiung, Taiwan; 4 Division of Cardiology, Department of Internal Medicine, E-Da Hospital, Kaohsiung, Taiwan; 5 Cardiovascular Division, Department of Internal Medicine, MacKay Memorial Hospital, Mackay Medical College, New Taipei City, Taiwan; 6 Institute of Clinical Medicine and Cardiovascular Research Center, National Yang-Ming University, Taipei, Taiwan; 7 Divison of Cardiology, Department of Medicine, Taipei Veterans General Hospital, Taipei, Taiwan; 8 Division of Cardiology, Heart Center, Cheng-Hsin General Hospital, and School of Medicine, National Yang-Ming University, Taipei, Taiwan; 9 Division of Cardiology, Department of Internal Medicine, Kaohsiung Medical University Hospital and Kaohsiung Medical University, Kaohsiung, Taiwan; 10 Division of Cardiovascular Medicine, China Medical University Hospital, Taichung, Taiwan; 11 Graduate Institute of Biomedical Sciences, China Medical University, Taichung, Taiwan; 12 Department of Cardiology, Buddhist Tzu-Chi General Hospital, Tzu-Chi University, Hualien, Taiwan; 13 Division of Cardiology, Department of Internal Medicine, National Taiwan University Hospital and National Taiwan University College of Medicine, Taipei, Taiwan; 14 Graduate Institute of Medical Education & Bioethics, College of Medicine, National Taiwan University, Taipei, Taiwan

**Keywords:** Prognostic markers, Coronary artery disease and stable angina

## Abstract

This study aimed to investigate the prognostic value of high-sensitivity creatine kinase-myocardial band or fraction (hsCK-MB) in comparison with other well-established biomarkers including heart type-fatty acid binding protein (H-FABP) and N-terminal pro-brain natriuretic peptide (NT-proBNP) in patients with stable coronary heart disease (SCHD). A total of 1,785 patients were enrolled and followed for 36 months. The primary outcome was all-cause mortality. The secondary outcomes included cardiovascular (CV) death, acute myocardial infarction (AMI), angina-related hospitalizations, and hospitalizations for heart failure. The all-cause mortality rate was significantly higher in the high hsCK-MB group compared to the low hsCK-MB group (4.64% vs. 1.88%, *p* = 0.0026). After adjusting for baseline covariates, there were no significant differences for the secondary outcomes. H-FABP (≥4.226 ng/mL) was the best predictor for all-cause mortality (HR = 2.68, 95% CI = 1.28–5.62, *p* = 0.009) and CV death (HR = 6.84, 95% CI = 1.89–22.14, *p* = 0.003). The high NT-proBNP group had a higher AMI-related hospitalization rate (HR = 1.91, 95% CI = 1.00–3.65, *p* = 0.05). Neither the addition of hsCK-MB to any other markers nor combinations of the three markers improved the prognostic significance of CV outcomes. In conclusion, hsCK-MB was an independent predictor for all-cause mortality but not CV outcomes in patients with SCHD. Combination of hsCK-MB, H-FABP and NT-proBNP failed to improve the prognostic power for all-cause mortality or CV outcomes.

## Introduction

In 2018, the World Health Organization reported that 17.9 million people die each year, an estimated 31% of all deaths worldwide, from cardiovascular diseases including coronary artery disease, cerebrovascular disease, rheumatic heart disease and other conditions. Furthermore, 85% of all cardiovascular deaths are due to heart attack and stroke, and 60% of cardiovascular deaths occur in low-income and middle-income countries. Diabetes mellitus, hypertension, dyslipidemia, tobacco use, physical inactivity and family history of premature coronary artery disease are known to increase the risk of cardiovascular diseases. However, traditional risk factors often lack sufficient sensitivity and specificity, and researchers have investigated the use of cardiac biomarkers such as creatine kinase-myocardial band or fraction (CK-MB), cardiac troponin I and troponin T, B-type natriuretic peptide and high-sensitivity C-reactive protein (hsCRP) to provide information beyond that of traditional risk factors. Cardiac troponins and CK-MB have been widely studied in acute coronary syndrome and periprocedural infarction after coronary intervention^1^, while B-type natriuretic peptide has been shown to be useful in the evaluation of heart failure. In the past decade, several novel biomarkers have been used to assess myocardial injury, for example, copeptin^2^, pentraxin-3^3^, heart type-fatty acid binding protein (H-FABP)^4^, procalcitonin^5^, vaspin^6^, myeloperoxidase^7^, and interleukin-6^8^, and the diagnostic and prognostic capabilities of these myocardial stretch, inflammatory and oxidative stress biomarkers in patient with stable coronary heart disease (SCHD) have been established. High-sensitivity troponin I and troponin T in patients with SCHD reflect the presence and extent of coronary artery disease, as well as the prognosis.

Creatine kinase has been used to diagnose myocardial infarction since the 1960s^9^. The separation of CK-MB, one of three creatine kinase isoenzymes which is found mostly in the heart, increased the accuracy of myocardial injury detection and has been routinely used to evaluate AMI since the 1970s^10^. CK-MB is normally undetectable or present at very low levels in normal individuals, however it can be detected in someone with a heart attack about 3–6 hours after the onset of acute chest pain, with a peak at 12–24 hours and then return to normal at about 48–72 hours. Creatine kinase and CK-MB have also been investigated with regards to the prognosis post percutaneous coronary interventions^11,12^, post coronary artery bypass surgery^13^, and acute pulmonary embolism^14^. Twenty years ago, CK-MB was considered to be the best biomarker to detect myocardial injury. However, cardiac troponin has since been proven to have higher sensitivity and specificity than CK-MB in stratifying the risk of acute coronary syndrome^15^, and unless the cardiac troponin assay is not available, CK-MB is no longer the recommended cardiac biomarker to evaluate myocardial infarction^16^. In addition, the American College of Cardiology/American Heart Association^17^ and European Society of Cardiology^18^ guidelines do not recommend the routine use of biomarkers in clinical practice. Furthermore, ESC guideline of heart failure and acute pulmonary embolism did not mention the clinical utility of CK-MB. Jaffe AS. et. al. discussed the controversy of post percutaneous coronary intervention biomarker increased imply an adverse prognosis. They suggested CK-MB no longer has a role in defining post-PCI injury because CK-MB is less sensitive and increases lag behind cardiac troponin. Cardiac troponin will provide all the prognostic information that is important with and without intervention^19^. However, the role of CK-MB in patients with SCHD is unclear, partly because few studies have used high-sensitivity CK-MB (hsCK-MB) measurements.

Hence, with data from patients with SCHD in a stable condition, we aimed to analyse evidence of major adverse cardiac events in patients with SCHD with high serum CK-MB levels using a hsCK-MB assay. We hypothesized that hsCK-MB might predict all-cause mortality and CV events. Furthermore, we aimed to assess whether using a combination of biomarkers can improve the risk stratification for mortality from CV causes beyond that of a model based only on established risk factors^20^. In this study, the use of hsCK-MB alone or in combination with other cardiac biomarkers with different mechanisms was evaluated to investigate whether the predictive value of hsCK-MB could be increased in patients with SCHD.

## Results

### Patients

A total of 1,785 SCHD patients from the National Taiwan Biosignature Research cohort study were enrolled and followed for 36 months or until a mortality or CV event. Among the 1,785 patients, 43 died during follow-up, including 21 CV deaths (Table 1). Approximately 45% of all CV events were related to angina-related hospitalization with revascularization.

**Table 1:** Cardiovascular events (n = 1785).

Events	Number of case (%)
All-cause mortality	43 (2.41%)
Cardiovascular mortality	21 (1.18%)
Acute myocardial infarction-related hospitalization	39 (2.18%)
Angina-related hospitalization with revascularization	216 (12.1%)
Angina-related hospitalization without revascularization	91 (5.01%)
Hospitalization for heart failure	63 (3.53%)

Except for a lower level of serum high-density lipoprotein cholesterol and lower left ventricular ejection fraction, the patients with a high level of hsCK-MB had significantly higher baseline rates of hypertension, systolic blood pressure, serum creatinine, history of type 2 diabetes mellitus, body mass index and fasting glucose than those with a low level of hsCK-MB (Table 2).

**Table 2:** Baseline characteristics of the patients with stable coronary heart disease, grouped by hsCK-MB levels.

	Total		hsCK-MB < 4.73 ng/mL n = 1440		hsCK-MB ≥ 4.73 ng/mL N = 345		p
	n	(%)	n	(%)	n	(%)	
Male sex	1514	84.82	1211	84.1	303	87.83	0.083
Hypertension	1155	64.71	916	63.61	239	69.28	0.048
Diabetes	674	37.76	514	35.69	160	46.38	0.0002
Smoking	1011	56.64	810	56.25	201	58.26	0.4984
Left main disease	33	1.85	23	1.6	10	2.9	0.1064
1-vessel disease	917	51.37	752	52.22	165	47.83	0.0825
2-vessel disease	231	12.94	192	13.33	39	11.3	0.3023
3-vessel disease	124	1.34	18	1.25	6	1.74	0.4784
		Median (IQR)		Median (IQR)		Median (IQR)	
Age, years	1785	63.16 (56.06–72.25)	1440	62.84 (56.05–72.21)	345	63.95 (56.32–72.77)	0.79
BMI (kg/m^2^)	1781	26.03 (23.83–28.58)	1438	26.00 (23.75–28.43)	343	26.17 (24.13–29.05)	0.05
Systolic BP, mmHg	1782	130 (119–28.58)	1439	130 (118–140)	343	132 (121–145)	0.001
Diastolic BP, mmHg	1782	74 (67–82)	1439	74 (67–82)	343	75 (67–84)	0.07
Glucose, mg/dL	1775	107 (95–133)	1432	106 (95–130)	343	115 (96–142)	0.0003
Haemoglobin g/dL	1704	13.8 (12.5–14.9)	1375	13.9 (12.6–14.9)	329	13.5 (11.8–14.8)	0.001
LDL-C, mg/dL	1780	91 (73–112)	1438	91 (74–113)	342	88.65 (72–107)	0.43
HDL-C, mg/dL	1777	40 (34.4–47.5)	1435	40 (34.9–48)	342	39 (33.9–46)	0.01
Serum creatinine, mg/dL	1781	1.03 (0.87–1.26)	1437	1.01 (0.85–1.21)	344	1.13 (0.91–1.60)	<0.001
eGFR, mL/min/1.73m^2^	1781	74.70 (59.48–91.46)	1437	76.55 (61.72–92.54)	344	66.4(44.50–86.26)	<0.001
LVEF (%)	1330	60 (52.4–68)	1034	60 (54–68)	269	57.2 (48.4–66)	0.01

Results are expressed as percentage or median (IQR).

BMI = body mass index; BP = blood pressure; eGFR=estimated glomerular filtration rate; HDL-C = high-density lipoprotein-cholesterol; LDL-C = low-density lipoprotein-cholesterol; NT-pro BNP = N-terminal pro-brain natriuretic peptide, LVEF = left ventricular ejection fraction, hsCK-MB = high-sensitivity creatine kinase-myocardial band.

### Predictive capabilities of individual biomarkers and their combinations for all-cause mortality

**Figure 1:**
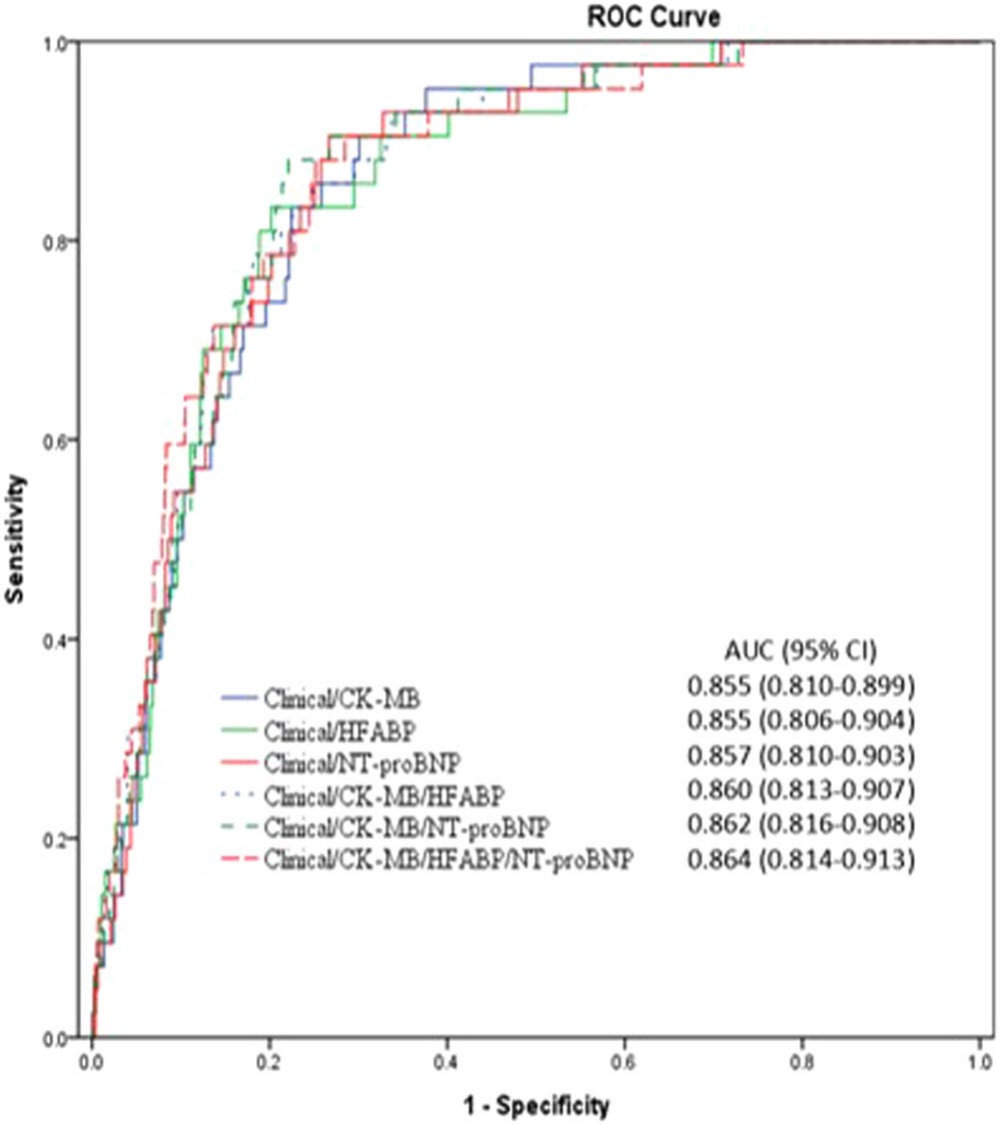
Receiver operating characteristic curve analysis with area under the curve, sensitivity and specificity of hsCK-MB, H-FABP and NT-proBNP and their combination in predicting all-cause mortality. (ROC = Receiver operating characteristic. hsCK-MB = high sensitivity creatine kinase-myocardial band, H-FABP = heart type-fatty acid binding protein, NT-proBNP = N-terminal pro-brain natriuretic peptide).

Using receiver operating characteristic (ROC) curve analysis, the cut-off values for CK-MB, H-FABP, and NT-proBNP are 4.730 ng/mL, 4.226 ng/mL, and 229.6 pg/mL, respectively. AUC of hsCK-MB, H-FABP and NT-proBNP are between 0.8 and 0.9, which indicate they are strong models to predict all-cause mortality (Fig. 1 and Table 3). The addition of H-FABP and NT-proBNP to hsCK-MB tended to raise the predictive power of individual hsCK-MB, and the combination of these three biomarkers tended to be the best model to predict all-cause mortality, while no statistical significance among these models were reached (*p* values were between 0.33 and 0.96).

**Table 3:** Identified characteristics of hsCK-MB, FABP and NT-proBNP and their combinations for all-cause mortality.

Logistic regression models	AUC (95% CI)	Cut off value	Sensitivity	specificity	p*
**Univariate models**					
hsCK-MB	0.855 (0.8102–0.8997)	4.73 ng/mL	0.372	0.811	—
H-FABP	0.8555(0.8064–0.9045)	4.225 ng/mL	0.628	0.786	0.9633
NT-proBNP	0.8572(0.8107–0.9036)	298.95 pg/mL	0.628	0.669	0.829
**Multivariate models**					
hsCK-MB + H-FABP	0.8603 (0.813–0.9077)				0.5346
hsCK-MB + NT-proBNP	0.8623 (0.8165–0.9081)				0.326
hsCK-MB + H-FABP + NT-proBNP	0.864 (0.814–0.913)				0.4576

^*^The *p* values compare to hsCK-MB model.

### Primary outcomes

**Figure 2:**
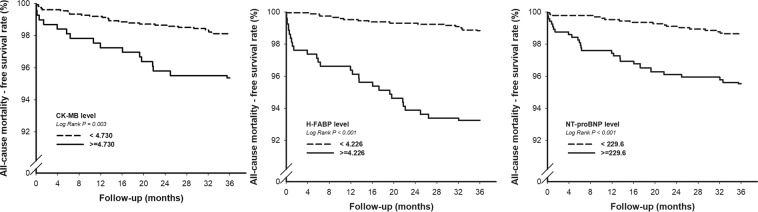
Kaplan-Meier survival curve analysis showing all-cause mortality in patients with serum hsCK-MB, H-FABP and NT-proBNP. (hsCK-MB = high sensitivity creatine kinase-myocardial band, H-FABP = heart type-fatty acid binding protein, NT-proBNP = N-terminal pro-brain natriuretic peptide).

After 36 months of follow-up, the high hsCK-MB (≥4.73 ng/mL) group had more than a two-fold higher rate of primary events than the low hsCK-MB group (4.64% vs. 1.88%, *p* < 0.0026) (Table 4). The Kaplan-Meier curves of the two groups were significantly separated from the beginning of the study to 36 months (Fig. 2a). In multivariate Cox proportional hazards analysis adjusted for age, sex, body mass index, estimated glomerular filtration rate, low-density lipoprotein cholesterol, high-density lipoprotein cholesterol, systolic blood pressure, smoking status, history of hypertension and diabetes mellitus, a high level of hsCK-MB was still an independent risk factor for all-cause mortality.

**Table 4:** Clinical outcomes after 36 months based on hsCK-MB levels.

	All (n = 1,785)	hsCK-MB < 4.73 ng/mL (n = 1440)	hsCK-MB ≥ 4.73 ng/mL (n = 345)	p
**Primary outcome**				
All-cause mortality, n (%)	43 (2.41%)	27(1.88%)	16 (4.64%)	0.0026
**Secondary outcome**				
CV mortality, n (%)	21 (1.18%)	12 (0.83%)	9 (2.16%)	0.006
AMI-related hospitalization	39 (2.18%)	28 (1.94%)	11 (3.19%)	0.1557
Angina-related hospitalization with revascularization	216 (12.1%)	176 (12.2%)	40 (11.59)	0.748
Angina-related hospitalization without revascularization	91 (5.01%)	69 (4.78%)	22 (6.38%)	0.2293
Hospitalization for heart failure	63 (3.53%)	43 (2.99%)	20 (5.80%)	0.011

CV = cardiovascular, hsCK-MB = high-sensitivity creatine kinase-myocardial band, AMI = acute myocardial infarction.

### Secondary outcomes

**Figure 3:**
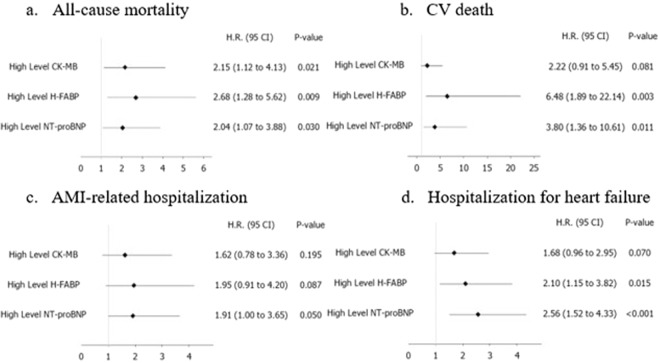
Comparisons of hsCK-MB, H-FABP and NT-proBNP in primary and secondary outcomes after adjusting for age, body mass index, sex, hypertension, type 2 diabetes mellitus, smoking, low-density lipoprotein, high-density lipoprotein and estimated glomerular filtration rate (hsCK-MB = high sensitivity creatine kinase-myocardial band, H-FABP = heart type-fatty acid binding protein, NT-proBNP = N-terminal pro-brain natriuretic peptide, CV = cardiovascular, AMI = acute myocardial infarction).

Of the 1,785 patients, 21 died of CV-related causes, including nine (2.16%) in the high hsCK-MB group and 12 (0.83%) in the low hsCK-MB group (Table 4 and see Supplementary Figure S1). In addition, the high hsCK-MB group had a significantly higher rate of hospitalization for heart failure (HHF) (5.80%) compared to the low hsCK-MB group (2.99%) (Table 4 and see Supplementary Figure S2). However, high hsCK-MB was not a significant predictor for any secondary outcome in multivariate Cox proportional hazards analysis (Fig. 3).

### Comparisons of hsCK-MB, H-FABP and NT-proBNP

The all-cause mortality within 30 days was low (7 deaths (0.39%)) (see Supplementary Table S3). During the 36-month study period, time-to-event data were evaluated with the use of Kaplan-Meier estimates of all serum markers, the number of total deaths was <1% in the first 4 months, it increased progressively with time. All of the 3 biomarkers (hsCK-MB, H-FABP and NT-proBNP) had very good predictive value for all-cause mortality, CV death and HHF. (Fig. 2, see Supplementary Figures S1 and S2). The difference of the survival rate between the patients with high and those with low baseline serum levels was similarly significant among the 3 markers. However, only NT-proBNP showed a significant predictive value for AMI-related hospitalizations (see Supplementary Figure S3).

After adjusting for age, body mass index, sex, hypertension, type 2 diabetes mellitus, smoking, low-density lipoprotein-cholesterol, high-density lipoprotein-cholesterol and estimated glomerular filtration rate, H-FABP (≥4.226 ng/mL) was the best predictor for all-cause mortality (HR 2.68, 95% CI 1.28–5,62, *p* = 0.009) and CV death (HR 6.84, 95% CI 1.89–22.14, *p* = 0.003), followed by NT-proBNP (≥229.6 pg/mL), and hsCK-MB (Fig. 3a,b).

In addition, the high NT-proBNP and high H-FABP groups had higher incidence rates of HHF, whereas hsCK-MB had no predictive value for HHF (Fig. 3d). Except for NT-proBNP (HR 1.91, 95% CI 1.00–3.65, p = 0.05), none of the cardiac biomarkers showed a significant prognostic power for AMI-related hospitalization (Fig. 3c).

### Multi-biomarker approach

**Figure 4:**
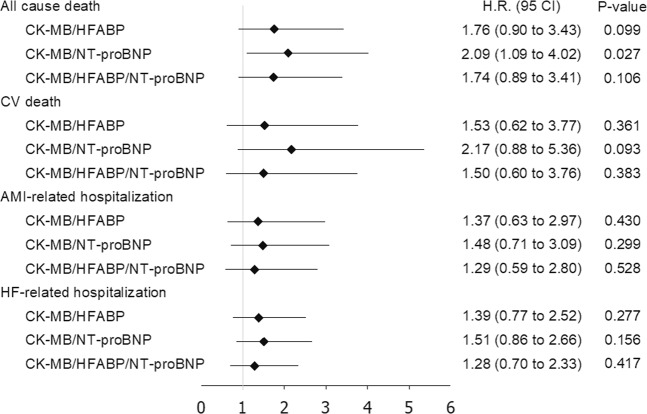
Multivariate logistic Cox-proportional regression analysis models for clinical outcomes in combinations of hsCK-MB with H-FABP and NT-proBNP. (hsCK-MB = high sensitivity creatine kinase-myocardial band, H-FABP = heart type-fatty acid binding protein, NT-proBNP = N-terminal pro-brain natriuretic peptide, CV = cardiovascular, AMI = acute myocardial infarction, HF = heart failure).

The add-on of hsCK-MB to H-FABP did not significantly improve the prognostic significance for CV outcomes (Fig. 4). Except for all-cause mortality, a combination of hsCK-MB with NT-proBNP actually weakened the prognostic impact on secondary outcomes compared to NT-proBNP alone. A combination of hsCK-MB/H-FABP/NT-proBNP did not improve the prognostic significance for both all-cause mortality (HR 1.74, CI 0.89–3.41, *p* = 0.106) and CV outcomes (see Supplementary Table S1).

### Propensity score matching

Some baseline variables were significantly different between high hsCK-MB group and low hsCK-MB group. Propensity score matching was performed with the following baseline variables: sex, hypertension and diabetes. Patients were 3:1 matched using the nearest-neighbour match. As compared with low hsCK-MB group, high hsCK-MB group had significant higher rates of all-cause mortality, CV mortality, AMI-related hospitalization, angina-related hospitalization without revascularization and hospitalization for heart failure (see Supplementary Table S2). In multivariable Cox proportional hazards analysis, high hsCK-MB group had a significantly higher rate of all-cause mortality (HR 2.08, CI 1.02–4.22, *p* = 0.043). There was no significant difference in all secondary outcomes between high and low hsCK-MB groups (see Supplementary Table S4). The result remained the same for the overall cohort analyses.

## Discussion

This study confirms that SCHD patients with a higher serum hsCK-MB level (≥4.730 ng/mL) have a higher risk of all-cause mortality. Compared to patients with acute coronary syndrome, this study is the first prospective cohort to establish the role of hsCK-MB in patients with SCHD. Several studies have reported the prognostic value of cardiac biomarkers after a percutaneous coronary intervention^11,12^ and coronary bypass surgery^13^. These studies consistently showed that even a small increase in CK-MB after the procedure was associated with significant late mortality. However, our study is unique because the cardiac biomarkers used for analysis were collected from individuals who had been stable on medical treatment for at least 1 month. After 36 months of follow-up, the analysis showed that the all-cause mortality rate in the high hsCK-MB group was about 2.5 times (4.64% vs. 1.88%, *p* < 0.0026) higher than that in the low hsCK-MB group. Moreover, approximately half of the deaths were due to cardiovascular diseases (21/43 deaths, 49%) in both the high hsCK-MB group (9/16, 56%) and low hsCK-MB group (12/27, 44%). Consequently, the high hsCK-MB group had a higher rate of CV death (2.16% vs. 0.83%, *p* = 0.006). However, in multivariate Cox proportional hazards analysis, the high hsCK-MB group showed only a trend toward significance in CV death (HR 2.22, 95% CI 0.91–5.45, *p* = 0.081). On the other hand, a higher serum hsCK-MB level (≥4.730 ng/mL) did not have predictive value for AMI-related hospitalizations in the patients with SCHD (3.19% vs 1.94%, p = 0.16). Furthermore, hsCK-MB could not predict angina-related hospitalizations, regardless of with or without revascularization.

In the 1970s, a German study investigated the serum activity of creatine kinase and CK-MB in 129 patients with various aetiologies of heart failure. CK-MB was found to be elevated in 19 patients, most of whom had inflammatory heart disease. However, they found no correlation between CK-MB activity in serum and the severity of heart failure^21^. A study from Turkey which included 162 individuals with (n = 104) or without (n = 58) symptoms and signs of HF examined the correlation of CK-MB and cardiac troponin I with disease severity^22^. The results showed that mean levels of CK-MB and cardiac troponin I in the symptomatic group with NYHA class III-IV were significantly higher than those in the asymptomatic group and symptomatic group with NYHA class I-II. The strength of the current study is that it enrolled a larger sample size (1,785 patients) in comparison to the other studies which investigated the relationship between CK-MB and heart failure. Consistent with the previous studies, we found borderline significance in the prognostic vale of HHF in patients with SCHD (HR 1.68, 95% CI 0.96 to 2.95, *p* = 0.07).

Biomarkers from different pathophysiological pathways may provide comprehensive prognostic information. In a prospective study of 1,034 patients, NT-proBNP showed long-term mortality predictive value in patients with stable coronary artery disease^23^. This myocardial stretch biomarker provides prognostic information above and beyond that provided by conventional CV risk factors and the degree of left ventricular systolic dysfunction. Held *et al*. found that interleukin 6, an inflammation biomarker, was independently associated with the risk of major adverse cardiac events, CV death and all-cause mortality, while hsCRP was not significantly associated with major adverse cardiac events, CV death or cancer death^8^. In our latest publication, we reported the prognostic significance of H-FABP in patients with SCHD after 24 months of follow-up^24^. The Kaplan-Meier curves of the two groups (H-FABP ≥ 4.226 ng/mL and <4.226 ng/mL) were more significantly separated from 24 months to 36 months (Fig. 2b). Therefore, we tried to clarify the prognostic values of hsCK-MB, H-FABP and NT-proBNP in patients with SCHD in this prospective study. The results demonstrated that H-FABP was the best predictor of all-cause mortality (HR 2.68, 95% CI 1.28–5,62, *p* = 0.009) and CV death (HR 6.84, 95% CI 1.89–22.14, *p* = 0.003), while NT-proBNP had the best performance in predicting HHF (HR 2.56, 95% CI 1.52–4.33, p < 0.001) and AMI-related hospitalizations (HR 1.91, 95% CI 1.00–3.65, p = 0.05). In addition, hsCK-MB was also a good predictor of all-cause mortality in the SCHD patients, but it was less powerful than H-FABP and NT-proBNP. Furthermore, hsCK-MB might have a less important role than H-FABP and NT-proBNP in predicting CV mortality. Except for NT-proBNP, none of the cardiac biomarkers showed prognostic value for AMI-related hospitalizations in the patients with SCHD.

In the current study, the all-cause mortality of the study patients, especially those with high baseline biomarker levels, was constantly reduced with time after enrollment. Furthermore, the high levels of baseline serum markers could be related to the onset of all-cause mortality within 1–4 months after enrollment, suggesting the significant and immediate prognostic impacts of these biomarkers. In fact, all the patients in this study were at very high risk since they had undergone coronary interventions in various durations before enrollment. The Kaplan-Meier estimates of all serum markers showed the relatively more deaths in the early period of enrolment. It is well known that the clinical process of CAD is dynamic, which may become unstable suddenly at any time either after a long stable period or just after enrollment. Besides, compared with those patients with low level, the patients with high baseline serum hsCK-MB level had more diabetes and hypertension, and lower serum HDL, hemoglobin and eGFR, which might partly contribute to the difference of all-cause mortality between high and low serum level groups. The prominent difference of event rate occurring in the first month implicated the good prognostic value of these serum markers in the early phase in the so-called “SCHD”^25^.

A small prospective study from India investigated the correlations between cardiac biomarkers (including B-type natriuretic peptide, Tn-I, TNF-α, and CK-MB) and the prognosis in 60 heart failure patients diagnosed according to the Framingham criteria^26^. The results showed that B-type natriuretic peptide had excellent predictive value while CK-MB played no role in this population. The results are consistent with our study, in that NT-proBNP and H-FABP were good prognostic biomarkers of HHF in the patients with SCHD, while hsCK-MB was not. Due to the baseline clinical variables were different between patients with high hsCK-MB and low hsCK-MB, for instance, sex, hypertension and diabetes. Propensity score matching was performed to minimize the bias. The result remained the same from the analyses with or without propensity score matching. It confirmed the prognostic significance of hsCK-MB.

Combination of multiple markers usually may enhance the risk assessment of CV disease. Kleber *et al*. developed a new Vienna and Ludwigshafen CAD (VILCAD) biomarker risk score, which is a composite of age, haemoglobin A1c, LVEF, heart rate, NT-proBNP, sex, renin, 25-OH vitamin D, and cystatin C to predict the future long-term prognosis of patients with SCHD^27^. Compared to the original VILCAD score which did not include novel biomarkers, the new VILCAD biomarker risk score represents a valuable tool for risk prediction in patients with SCHD. Vimal *et al*. used a simple multi-marker risk stratification approach which grouped B-type natriuretic peptide, hsCRP and cardiac troponin, however, this integrated assessment did not improve the prognostic information in patients who underwent percutaneous coronary intervention^28^. In our study, the add-on of hsCK-MB to H-FABP and NT-proBNP even reduced the prognostic value of CV outcomes compared to the results of single biomarkers. The combination of hsCK-MB/H-FABP/NT-proBNP also failed to improve the prognostic power for CV outcomes. Taken together, these results suggest that neither traditional risk factors nor novel biomarkers alone are good tools for all risk prediction, but rather that they need to be used in various combination with clinical parameters for different conditions. Single best marker or sequential markers policy might be more cost-effective under such circumstance. Further investigations with different markers in a larger study population are warranted.

The limitations of this study are as follows. The patient enrolment criteria and the specified protocol for clinical follow-up could not fully eliminate selection bias^29^. This hospital-based design might have been affected by environmental exposure to cardiovascular disease risk factors and other such geographic variations^30^. Although the patients were regularly monitored and recorded as being stable during out-patient visits, the cardiologists may have modified the patient’s medication. Therefore, the prescriptions might have affected the clinical outcome^31^. A minor number of deaths lessened the statistical power of this study. Finally, based on the Kaplan-Meier curves, there are many deaths within 1 month of the follow-up, we did not analyze the time interval between the enrollments and prior coronary interventions, as well as the indication of prior coronary interventions in this study. In summary, the biomarker hsCK-MB was a significant predictor but not the best one to predict all-cause mortality of the patients with SCHD at 36 months. In between the biomarkers, H-FABP was the best predictor for all-cause and CV mortality, while NT-proBNP had the best performance in predicting HHF and AMI-related hospitalizations. There was significant difference in the biomarker levels and roles among different outcome measures. However, multi-markers approach failed to improve the prognostic power for CV outcomes in the patients with SCHD, and the additive value of hsCK-MB as well as a multi-biomarker model seems limited.

## Methods

### Study population

This National Taiwan Biosignature Research study involved nine Taiwanese medical centers with a cohort of SCHD patients (aged >20 years)^29^. SCHD was defined as the patients who had undergone percutaneous coronary intervention remained steady for more than 30 days. The exclusion criteria were patients who had a prior hospitalization within 90 days before enrolment, who had a life expectancy of less than 6 months, or were unable or unwilling to receive follow-up.

### Baseline clinical and data collection

The physicians and nurses gathered data when possible following enrolment. Baseline characteristics including sex, age, hypertension, type 2 diabetes mellitus, dyslipidemia, smoking status, body mass index, number of stenotic coronary arteries, and biochemical data including renal function and lipid profile were recorded at each hospital.

### Biomarker assays

Cardiovascular diseases serum markers were measured using an EMD Millipore MILLIPLEX MAP Human Cardiovascular diseases Panel 1 Magnetic Bead kit (Millipore, Inc., MO, USA) with the Luminex xMAP® platform (Millipore, Inc.), which offers the advantage of ideal speed and sensitivity. The minimum detectable concentrations of hsCK-MB, H-FABP and N-terminal pro-brain natriuretic peptide (NT-proBNP) were 43.0 pg/mL, 24.9 pg/mL and 18.8 pg/mL, respectively.

### Clinical follow-up

Questionnaires and blood samples were collected from the patients every quarter in the first year, and bi-annually from the second year after enrolment. The primary outcome was all-cause mortality, and the secondary outcomes included CV death, AMI, angina-related hospitalization with revascularization, angina-related hospitalization without revascularization, and HHF. All the clinical events were affirmed by the Taiwanese Welfare Data Science Centre.

The relevant health authorities, independent ethics committees and institutional review boards approved this study which also complied with the Declaration of Helsinki. Signed consent forms were obtained from all of the patients enrolled in the study.

### Statistical analysis

SAS version 9.4 for Windows was used. We showed the continuous variables in the median range and qualitative variables shown as absolute frequency. Comparisons of continuous variables between groups were performed using ANOVA or the Mann-Whitney *U* test. The primary and secondary outcomes were described as overall percentages and expressed as means of proportions with a 95% confidence interval (CI). Calculations of events and survival rates were identified using the Kaplan-Meier method. Hazard ratios (HRs) for the regression of Cox proportional hazards were used, along with the corresponding standard error, 95% CI, and *p* value. The multi-variate analysis incorporated the independent baseline variables with a p value <0.05 in the univariate analysis. In all tests, the two-tailed alpha significance level was 0.05.

The ability of biomarkers to discriminate outcomes was assessed using receiver operating characteristic (ROC) curve analysis. The values of area under the curve (AUC) of the ROC curve over 0.7 indicate a favorable model, values over 0.8 indicate a strong model and values between 0.9 and 1 indicate a very strong model. The comparison of AUC among the biomarkers and models was conducted by using the DeLong’s test. The sensitivity and specificity of the biomarkers were also analyzed. The optimal cutoff values of each biomarker were determined by the maximum Youden index (Youden index = sensitivity + specificity −1). Each marker was separated into high and low serum level groups based on the optimal cutoff value. Then, with the use of multivariate Cox proportional hazards analysis adjustment, the prognostic values of each high and low serum biomarker groups were taken out for comparison. Comparisons of characteristics and CV outcomes were made between the patients with high and low levels of the biomarkers. Additional propensity score matching was performed.

## Supplementary information

Supplementary information
