# Supplementary material for: Potential impacts of high-sensitivity creatine kinase-MB on long-term clinical outcomes in patients with stable coronary heart disease
Source: Sci Rep. 2020 Mar 27;10:5638. doi: 10.1038/s41598-020-61894-3 (PMC7101408; doi:10.1038/s41598-020-61894-3)
Supplement: Supplementary file 1 — Supplementary information [file 41598_2020_61894_MOESM1_ESM.docx]

**Potential impacts of high-sensitivity creatine kinase-MB on long-term clinical outcomes in patients with stable coronary heart disease**

Yen-Wen Wu^a,b^, Sing Kong Ho^a^ , Wei-Kung Tseng^c,d^, Hung-I Yeh^e^, Hsin-Bang Leu^f,^[^g^](file:///C:\Users\Yuchen\Google%20雲端硬碟\CKMB\CK-MB%20manuscript%2020190610Ho.docx#g), Wei-Hsian Yin^h^, Tsung-Hsien Lin^i^, Kuan-Cheng Chang^j,k^, Ji-Hung Wang^l^, , Chau-Chung Wu^m,n[*](file:///C:\\Users\\Yuchen\\Google%20雲端硬碟\\CKMB\\CK-MB%20manuscript%2020190610Ho.docx#corespond)^, Jaw-Wen Chen^f,g*^

^a^Cardiology Division of Cardiovascular Medical Center, Far Eastern Memorial Hospital, New Taipei City, Taiwan

^b^National Yang-Ming University School of Medicine, Taipei, Taiwan

^c^Department of Medical Imaging and Radiological Sciences, I-Shou University, Kaohsiung, Taiwan

^d^Division of Cardiology, Department of Internal Medicine, E-Da Hospital, Kaohsiung, Taiwan

^e^Cardiovascular Division, Department of Internal Medicine, MacKay Memorial Hospital, Mackay Medical College, New Taipei City, Taiwan

^f^Institute of Clinical Medicine and Cardiovascular Research Center, National Yang-Ming University, Taipei, Taiwan

^g^Divison of Cardiology, Department of Medicine, Taipei Veterans General Hospital, Taipei, Taiwan

^h^Division of Cardiology, Heart Center, Cheng-Hsin General Hospital, and School of Medicine, National Yang-Ming University, Taipei, Taiwan

^i^Division of Cardiology, Department of Internal Medicine, Kaohsiung Medical University Hospital and Kaohsiung Medical University, Kaohsiung, Taiwan

^j^Division of Cardiovascular Medicine, China Medical University Hospital, Taichung, Taiwan

^k^Graduate Institute of Biomedical Sciences, China Medical University, Taichung, Taiwan

^l^Department of Cardiology, Buddhist Tzu-Chi General Hospital, Tzu-Chi University, Hualien, Taiwan

^m^Division of Cardiology, Department of Internal Medicine, National Taiwan University Hospital and National Taiwan University College of Medicine, Taipei, Taiwan

^n^Graduate Institute of Medical Education & Bioethics, College of Medicine, National Taiwan University, Taipei, Taiwan

**Yen-Wen Wu and Sing Kong Ho contributed equally as first co-authors to this article.**

**Chau-Chung Wu and Jaw-Wen Chen contributed equally to this article.**

***For correspondence and reprints contact:**

**Chau-Chung Wu, MD, PhD**

Department of Internal Medicine (Cardiology Section), National Taiwan University Hospital, No. 7, Chung-Shan South Road, Taipei 100, Taiwan

Tel: +886-2-2312-3456 ext. 88560; Fax: +886-2-2321-7485

Email: [chauchungwu@ntu.edu.tw](mailto:chauchungwu@ntu.edu.tw)

**Jaw-Wen Chen, MD**

Division of Clinical Research, Department of Medical Research;

and Division of Cardiology, Department of Medicine,

Taipei Veterans General Hospital, Taiwan, R.O.C.

201, Section 2, Shih-Pai Road, Taipei, Taiwan, R.O.C.

Tel: +886-2-28757434 ext. 226

Fax: +886-2-28711601, +886-2-28757435

E-mail: [jwchen@ym.edu.tw](mailto:jwchen@ym.edu.tw)

Table S1 Multivariate logistic Cox-proportional regression analysis models for clinical outcomes with combinations of hsCK-MB, H-FABP and NT-proBNP.

|  | **hsCK-MB + H-FABP + NT-proBNP** | |
| --- | --- | --- |
|  | **HR (95% CI)** | ***P*** |
| All-cause mortality | 1.74 (0.89 to 3.41) | 0.106 |
| CV mortality, n (%) | 1.50 (0.60 to 3.76) | 0.383 |
| AMI-related hospitalization | 1.29 (0.59 to 2.80) | 0.528 |
| Hospitalization for heart failure | 1.28 (0.70 to 2.33) | 0.417 |

hsCK-MB=high sensitivity creatine kinase-myocardial band, H-FABP=heart type-fatty acid binding protein, NT-proBNP=N-terminal pro-brain natriuretic peptide, CV=cardiovascular, AMI=acute myocardial infarction

Table. S2 Clinical outcomes after 36 months based on hsCK-MB levels with propensity score match (3:1).

|  | All  (n=1,340) | hsCK-MB <4.73 ng/mL (n=1005) | hsCK-MB ≥4.73 ng/mL (n=335) | *p* |
| --- | --- | --- | --- | --- |
| **Primary outcome** | | | | |
| All-cause mortality, n (%) | 34 | 19(1.89%) | 15(4.48%) | 0.009 |
| **Secondary outcome** | | | | |
| CV mortality, n (%) | 18 | 9(0.10%) | 9(2.69%) | 0.0361 |
| AMI-related hospitalization | 32 | 21(2.09%) | 11(3.28%) | 0.0111 |
| Angina-related hospitalization with revascularization | 68 | 46(4.58%) | 22(6.57%) | 0.8043 |
| Angina-related hospitalization without revascularization | 164 | 126(12.54%) | 38(11.34%) | 0.0168 |
| Hospitalization for heart failure | 48 | 30(2.99%) | 18(5.37%) | 0.0051 |

CV=cardiovascular, hsCK-MB=high-sensitivity creatine kinase-myocardial band, AMI=acute myocardial infarction

Table S3 All-cause mortality within 30 days of follow-up based on high or low serum levels of biomarkers and CV or non-CV cause of deaths.

|  | Low serum level group  n=1440 | | | High serum level group  n=345 | | |
| --- | --- | --- | --- | --- | --- | --- |
|  | total | CV death | Non-CV death | total | CV death | Non-CV death |
| hsCK-MB | 4 (0.28%) | 2 | 2 | 3 (0.86%) | 3 | 0 |
| **H-FABP** | 0 (0%) | 0 | 0 | 7 (2.03%) | 5 | 2 |
| NT-proBNP | 2 (0.14%) | 1 | 1 | 5 (1.45%) | 4 | 1 |

Table S4 Multivariable logistic Cox-proportional regression analysis models for clinical outcomes with propensity score match (1:3).

|  | **hsCK-MB** | **H-FABP** | **NT-proBNP** |
| --- | --- | --- | --- |
|  | **HR (95% CI)**  ***p* value** | | |
| All-cause mortality | 2.08 (1.02 to 4.22)  0.043 | 2.83 (1.22 to 6.55)  0.015 | 1.71 (0.82 to 3.55)  0.152 |
| CV mortality | 2.27 (0.88 to 5.87)  0.091 | 6.54 (1.66 to 25.73)  0.007 | 2.92 (1.01 to 8.45)  0.048 |
| AMI-related hospitalization | 1.55 (0.73 to 3.31)  0.256 | 2.41 (1.08 to 5.38)  0.032 | 2.56 (1.24 to 5.28)  0.011 |
| Angina-related hospitalization with revascularization | 0.85 (0.59 to 1.24)  0.409 | 1.13 (0.76 to 1.67)  0.557 | 1.28 (0.93 to 1.77)  0.131 |
| Angina-related hospitalization without revascularization | 1.42 (0.84 to 2.40)  0.190 | 0.95 (0.51 to 1.76)  0.861 | 1.30 (0.79 to 2.14)  0.311 |
| Hospitalization for heart failure | 1.67 (0.90 to 3.08)  0.102 | 1.99 (1.02 to 3.89)  0.044 | 2.18 (1.20 to 3.95)  0.011 |

hsCK-MB=high sensitivity creatine kinase-myocardial band, H-FABP=heart type-fatty acid binding protein, NT-proBNP=N-terminal pro-brain natriuretic peptide, hsCRP=high sensitivity C-reactive protein, CV=cardiovascular, AMI=acute myocardial infarction

Figure S1 Kaplan–Meier survival curves analysis showing CV death in patients with higher and lower serum hsCK-MB, H-FABP and NT-proBNP.


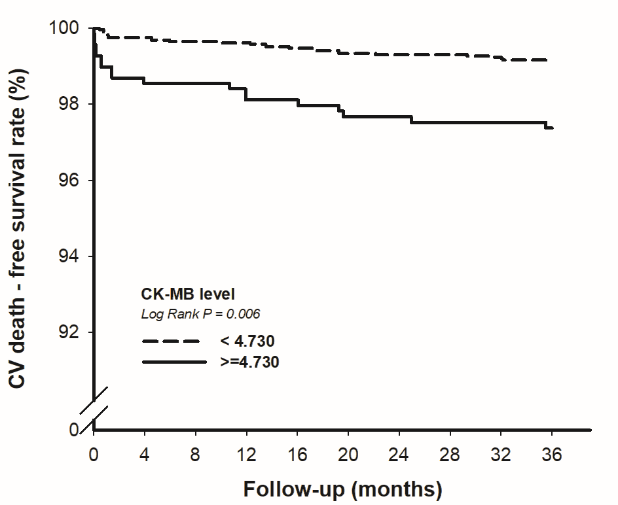

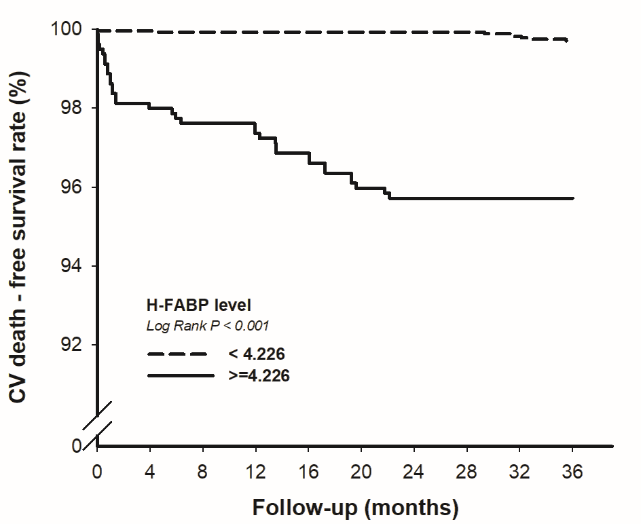

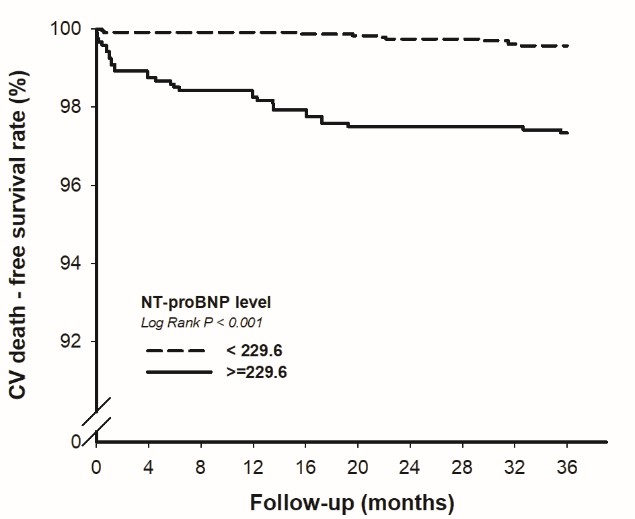


hsCK-MB= high sensitivity creatine kinase-myocardial band, H-FABP=heart type-fatty acid binding protein, NT-proBNP= N-terminal pro-brain natriuretic peptide*,* CV=cardiovascular

Figure S2 Kaplan–Meier survival curves analysis showing hospitalization for heart failure in patients with higher and lower serum hsCK-MB, H-FABP and NT-proBNP


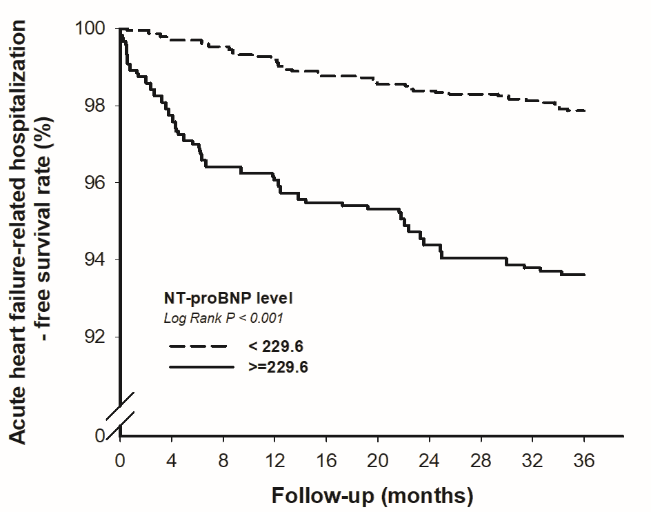

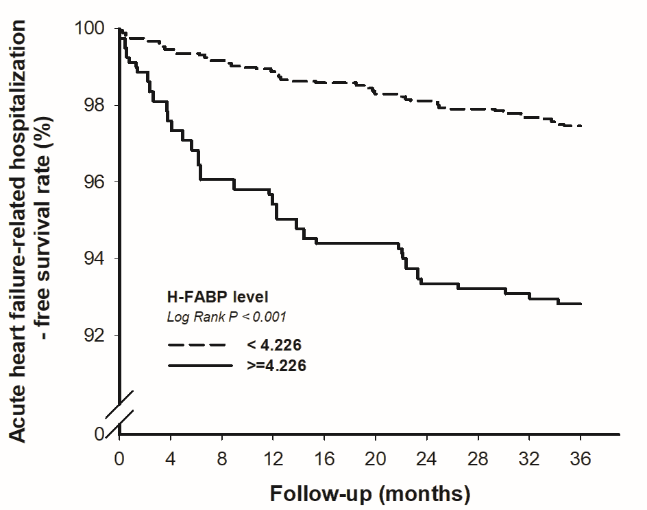

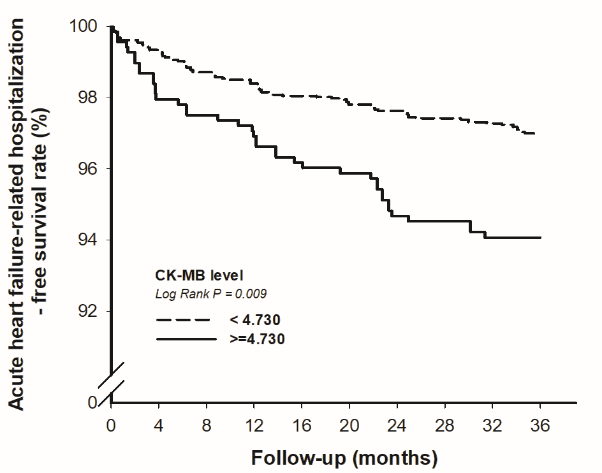


hsCK-MB= high sensitivity creatine kinase-myocardial band, H-FABP=heart type-fatty acid binding protein, NT-proBNP= N-terminal pro-brain natriuretic peptide

Figure S3 Kaplan–Meier survival curves analysis showing acute myocardial infarction-related hospitalization in patients with higher and lower serum hsCK-MB, H-FABP and NT-proBNP.


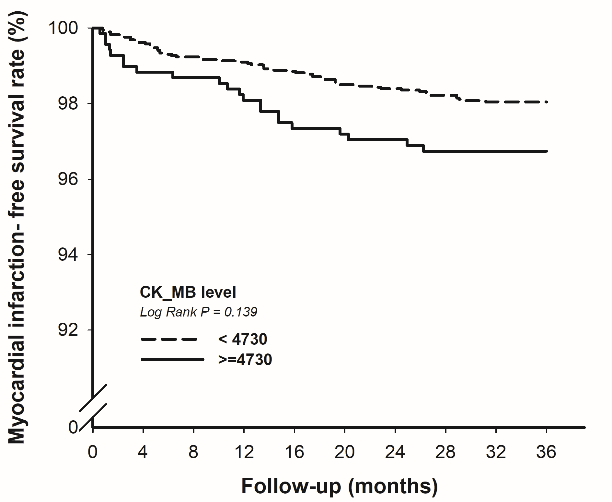

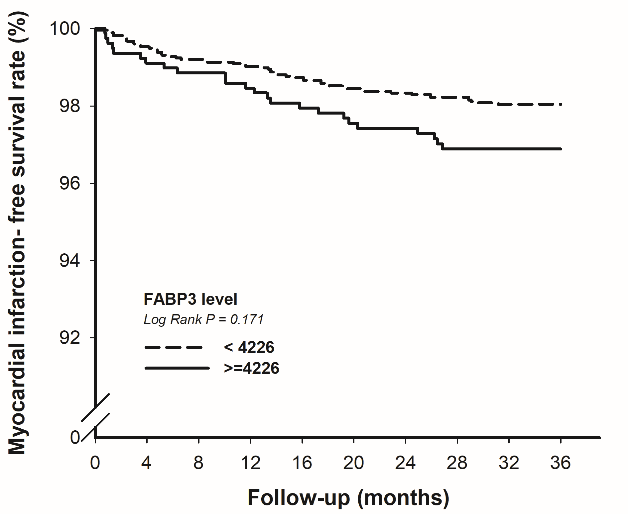


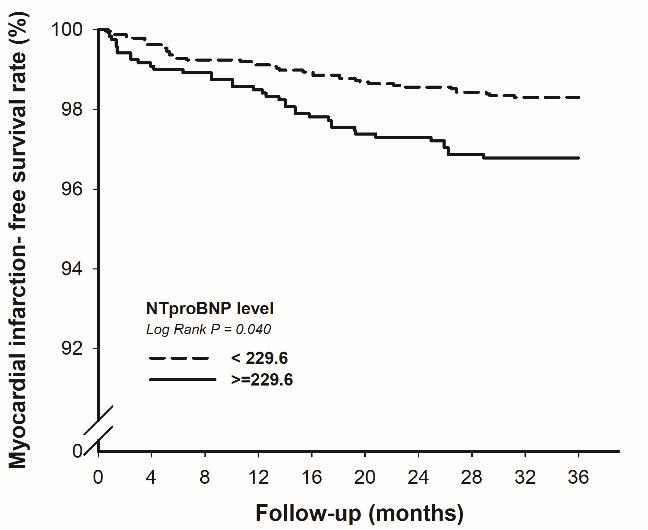


hsCK-MB*=* high sensitivity creatine kinase-myocardial band*,* H-FABP=heart type-fatty acid binding protein, NT-proBNP= N-terminal pro-brain natriuretic peptide
